# Ecological drivers of song evolution in birds: Disentangling the effects of habitat and morphology

**DOI:** 10.1002/ece3.3760

**Published:** 2018-01-13

**Authors:** Elizabeth Perrault Derryberry, Nathalie Seddon, Graham Earnest Derryberry, Santiago Claramunt, Glenn Fairbanks Seeholzer, Robb Thomas Brumfield, Joseph Andrew Tobias

**Affiliations:** ^1^ Museum of Natural Science and Department of Biological Sciences Louisiana State University Baton Rouge LA USA; ^2^ Department of Ecology and Evolutionary Biology Tulane University New Orleans LA USA; ^3^ Department of Ecology and Evolutionary Biology University of Tennessee Knoxville TN USA; ^4^ Department of Zoology Edward Grey Institute University of Oxford Oxford UK; ^5^ Department of Natural History Royal Ontario Museum Toronto ON Canada; ^6^ Department of Ornithology American Museum of Natural History New York NY USA; ^7^ Department of Life Sciences Imperial College London London UK

**Keywords:** acoustic adaptation, biomechanical constraints, bird song, Furnariidae, speciation, stochasticity, trade‐offs

## Abstract

Environmental differences influence the evolutionary divergence of mating signals through selection acting either directly on signal transmission (“sensory drive”) or because morphological adaptation to different foraging niches causes divergence in “magic traits” associated with signal production, thus indirectly driving signal evolution. Sensory drive and magic traits both contribute to variation in signal structure, yet we have limited understanding of the relative role of these direct and indirect processes during signal evolution. Using phylogenetic analyses across 276 species of ovenbirds (Aves: Furnariidae), we compared the extent to which song evolution was related to the direct influence of habitat characteristics and the indirect effect of body size and beak size, two potential magic traits in birds. We find that indirect ecological selection, via diversification in putative magic traits, explains variation in temporal, spectral, and performance features of song. Body size influences song frequency, whereas beak size limits temporal and performance components of song. In comparison, direct ecological selection has weaker and more limited effects on song structure. Our results illustrate the importance of considering multiple deterministic processes in the evolution of mating signals.

## 1 INTRODUCTION

Differences in mating signals among related lineages have important functional consequences for mate choice and species recognition (Coyne & Orr, 2004; Mayr, 1963). Understanding how such differences arise is therefore a key step in explaining the evolution of reproductive isolation and ultimately speciation (Lande, 1981). Much of the debate about mating signal diversification has centered on the role of sexual selection and social competition (Grether, Losin, Anderson, & Okamoto, 2009; Seddon et al., 2013; West‐Eberhard, 1983) and the extent to which these socially mediated factors interact with ecological selection (Boughman, 2002; Sobel, Chen, Watt, & Schemske, 2009; Wilkins, Seddon, & Safran, 2013). However, although the role of ecology mediated by habitat differences was once considered to be relatively straightforward, recent work has highlighted increasing disagreement about the ecological mechanisms underlying signal diversification (Servedio, Doorn, Kopp, Frame, & Nosil, 2011; Wilkins et al., 2013).

A prominent issue is that ecological diversity drives the evolution of mating signals in two distinct ways. First, differences in the transmission properties of habitats can lead to divergence in mating signals as a result of direct habitat‐dependent selection for effective signal transmission (Morton, 1975), a process termed “sensory drive” (Endler, 1992). Second, ecological selection can influence mating signals indirectly by causing divergence in traits related to signal production and modification (Endler, 1993), such as body size (Gil & Gahr, 2002) and beak size (Podos & Nowicki, 2004b) in birds. Such traits have been termed “magic traits” because under divergent ecological selection, they give rise “as if by magic” to signal divergence, and ultimately nonrandom mating, resolving a long‐standing difficulty in models of ecological speciation (Gavrilets, 2004; Thibert‐Plante & Gavrilets, 2013).

Direct and indirect ecological selection on mating signals are not mutually exclusive and both have been demonstrated individually across a wide array of taxa and signal modalities (Boughman, 2002; Cummings, 2007; Hausberger, Black, & Richard, 1991; Leal & Fleishman, 2004; Palacios & Tubaro, 2000; Podos, 2001; Seddon, 2005; Slabbekoorn & Smith, 2002b). Previous studies on sensory drive have controlled for the effect of morphology in order to focus on the ecological trait of interest (e.g., Slabbekoorn & Smith, 2002b; Wiley, 1991) or controlled for environmental variation to focus on morphology (e.g., Kirschel, Blumstein, & Smith, 2009). However, few studies have considered the relative roles of direct and indirect ecological selection on signal structure (e.g., Mason & Burns, 2015; Seddon, 2005). In the following sections, we outline evidence for direct and indirect ecological selection on acoustic mating signals and then address their relative contribution and potential interaction in the evolution of birdsong.

### 1.1 Sensory drive

Selection should favor signal traits that optimize transmission of information from signaler to receiver (Endler, 1993). In long‐distance signals, the physical properties of habitats may affect sound transmission, leading to the adaptation of signals to specific environments (Morton, 1975). For example, acoustic signals in forests are subject to scattering effects by vegetation, whereas in more open habitats, they are affected by wind (Richards & Wiley, 1980; Wiley & Richards, 1978). Consequently, acoustic signals of forest species tend to have slower pace, lower frequencies (e.g., Morton, 1975; Ryan & Brenowitz, 1985; Wiley, 1991), and more pure tones (e.g., Richards & Wiley, 1980; Wiley, 1991; Wiley & Richards, 1978) than those of species found in open, grassland habitats. This form of sensory drive (often termed “acoustic adaptation”) has shaped the evolution of bird song in most species examined (reviewed by Slabbekoorn & Smith, 2002a). However, a meta‐analysis found support for habitat shaping spectral rather than temporal features of song, and the overall effect of habitat on signal structure was small (Boncoraglio & Saino, 2007).

### 1.2 Magic traits

Animal signals are subject to indirect sources of selection because they are produced by traits with multiple functions (Nowicki, Westneat, & Hoese, 1992). For example, divergent ecologies can select for differences in body size (Grant, 1968), which in turn places limits on the fundamental frequency of sounds (Wallschäger, 1980). Because the fundamental frequency of birdsong is determined by the vibrating frequency of the syringeal membrane (Nowicki & Marler, 1988), larger birds tend to produce lower frequency song (Palacios & Tubaro, 2000; Ryan & Brenowitz, 1985; Tubaro & Mahler, 1998).

Similarly, the beak is under strong selection in the context of foraging and food manipulation (Grant, 1968; Herrel, Podos, Huber, & Hendry, 2005) and is used in coordination with vocal tract movements to modify sound (Goller, Mallinckrodt, & Torti, 2004; Westneat, Long, Hoese, & Nowicki, 1993). This has particular relevance to the widespread trade‐off between rates of sound production and the frequency bandwidth of sounds (Derryberry et al., 2012; Podos, 1997). This trade‐off has a triangular distribution because sounds produced at a slow rate can have a wide or a narrow frequency bandwidth, whereas as the rate of sound production increases, frequency bandwidth narrows. Ability to perform this trade‐off (i.e., “vocal performance”) may be affected by beak size through trade‐offs in jaw biomechanics, namely between maximal force and velocity (Herrel, Podos, Vanhooydonck, & Hendry, 2008; Herrel et al., 2005) and/or between torque and angular velocity (Palacios & Tubaro, 2000). In support of this hypothesis, morphological adaptation is associated with variation in song structure and performance capabilities in many species of birds (Badyaev, Young, Oh, & Addison, 2008; Ballentine, 2006; Derryberry, 2009; Derryberry et al., 2012; Huber & Podos, 2006; Podos, 2001; Seddon, 2005; Tobias et al., 2014).

### 1.3 Relative roles of sensory drive and magic traits

Despite extensive research on both direct and indirect sources of ecological selection on bird song, we are only aware of two studies considering both possibilities in tandem (Mason & Burns, 2015; Seddon, 2005). The first study demonstrated that indirect and direct selection both played a role in the evolution of song in antbirds (Thamnophilidae) (Seddon, 2005), although a species‐level molecular phylogeny was not available. More recently, Mason et al. (2017) found that body size was more important than habitat in the evolution of song in tanagers (Thraupidae), but no information was available regarding beak size. Thus, we still have only a limited understanding of the relative roles of these mechanisms, partly because comprehensive information on phylogenetic relationships, signal design, morphology, and ecology are rarely available for large radiations.

In this study, we use phylogenetic comparative techniques to assess the relative roles of direct and indirect ecological selection on song diversification across 285 species of ovenbirds (Furnariidae), a diverse clade with comprehensive data on phylogenetic relationships, morphology, and song (Derryberry et al., 2012; Tobias et al., 2014). Ovenbirds are an ideal system because they exhibit high diversity in both habitat preferences and morphological characters associated with feeding (Claramunt, 2010; Marantz, Aleixo, Bevier, & Patten, 2003; Raikow, 1994; Remsen, 2003; Tubaro, Lijtmaer, Palacios, & Kopuchian, 2002). Moreover, in common with other tracheophone suboscine passerines (Tobias & Seddon, 2009; Tobias et al., 2012; Touchton, Seddon, & Tobias, 2014), their songs appear to be innate with song learning limited or absent. This minimizes the effect of cultural processes on song evolution (Mason et al., 2017; Weir & Wheatcroft, 2011) and means that ovenbird songs are relatively simple and amenable to acoustic analysis (Tobias et al., 2012).

We used model comparison to assess the relative roles of direct ecological selection via sensory drive and indirect ecological selection via magic traits. To test the role of sensory drive, we predicted that species found in more closed habitats would produce songs at slower rates, with lower frequency characteristics and narrower bandwidths. To test the “magic traits” hypothesis, we predicted that species with larger body size would produce lower frequency songs (Nowicki & Marler, 1988) and that species with larger beaks would produce songs at a slower pace, narrower bandwidth, and lower vocal performance (Huber & Podos, 2006; Podos, 2001). Finally, several studies have highlighted the prominent role of stochasticity in explaining signal variation within and between species (Irwin, Thimgan, & Irwin, 2008; McCracken & Sheldon, 1997; Mundinger, 1982; Price & Lanyon, 2002), and thus, song divergence may simply be related to evolutionary time since speciation (Pagel, 1999; Tobias et al., 2010). Combining data on habitat, morphology, and phylogenetic relationships allowed us to test the relative influence of sensory drive and magic traits against this stochastic null model.

## 2 MATERIALS AND METHODS

### 2.1 Study species

**Figure 1:**
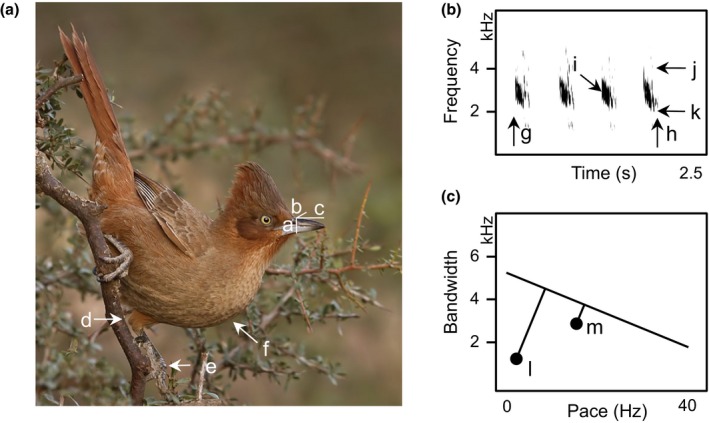
Phenotypic traits of ovenbirds. Exemplar data used in this study, illustrated for a single species (Brown cacholote, *Pseudoseisura lophotes*). (A) Morphological measurements collected from museum specimens, including beak depth (a), width (b) and length (c), tarsus length (d to e), and body mass (f). (B) Spectrogram of song segment indicating acoustic traits measured, including duration (g–h), pace (song duration/number of notes), peak frequency (i), maximum frequency (j), minimum frequency (k), and frequency bandwidth (j–k). (C) Frequency bandwidth plotted as a function of pace with the upper‐bound regression for the Furnariidae (*y* = −79.374*x* + 5066.2) and the orthogonal distance (vocal deviation) for a song of *P. lophotes* (l), which has comparatively lower vocal performance than song of many other ovenbird species, for example, *Schizoeaca fuliginosa* (m). Photograph by Mario Fiorucci; song file downloaded from www.xeno-canto.org (XC151258)

**Figure 2:**
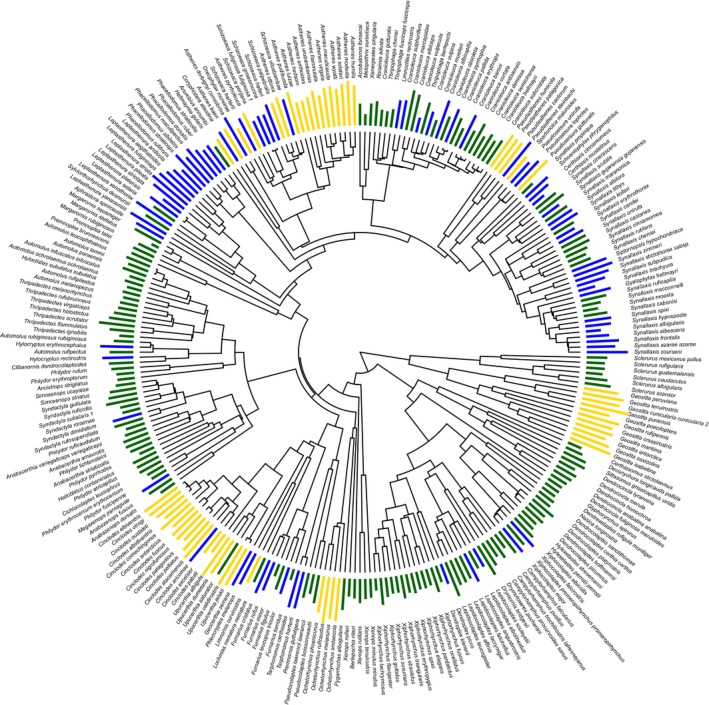
Phylogenetic hypothesis and habitat preferences for the ovenbird radiation. Colored bars show two different types of habitat data associated with tree tips. Height of bars indicates value of Environmental PC1 extracted from geographical range polygons; color‐coding of bars reflects habitat type categories generated from the literature (closed habitats = green; semi‐open habitats = blue; open habitats = yellow)

Ovenbirds (Furnariidae) are insectivorous passerine birds occurring in nearly every terrestrial habitat throughout Central and South America. The radiation is unusually diverse, comprising 69–74 genera and approximately 295 extant species (Remsen et al., 2011). We followed the classifications of Marantz et al. (2003) and Remsen (2003), including more recent modifications modified according to more recent studies (Chesser, Claramunt, Derryberry, & Brumfield, 2009; Claramunt, Derryberry, Chesser, Aleixo, & Brumfield, 2010; Derryberry, Claramunt, Chesser, et al., 2010; Derryberry, Claramunt, O'Quin, et al., 2010; Remsen et al., 2011). We included four data sets within this study: vocal, morphological, environmental, and genetic (Figures 1 and 2). Our genetic data set sampled 285 of the 295 recognized species and all recognized genera (Derryberry et al., 2011). Our vocal, morphological, and environmental data sets comprised complete data on 276 of these 285 ovenbird taxa, or ~94% of recognized species diversity.

### 2.2 Song data

Many species of ovenbirds have a wide vocal repertoire including calls and so‐called loudsongs—a consistently patterned, multiple‐note vocalization typically repeated at regular intervals (Willis, 1967). Observational studies on ovenbirds suggest that loudsongs function in territory defense, mate attraction, and pair bonding (Ippi, Vasquez, Van Dongen, & Lazzoni, 2011; Kratter & Parker, 1997; Roper, 2005; Zimmer, Robbins, & Kopuchian, 2008), in common with other tracheophone suboscine birds in which function has been tested experimentally (Tobias, Gamarra‐Toledo, Garcia‐Olaechea, Pulgarin, & Seddon, 2011). As tracheophone suboscine loudsongs are therefore functionally equivalent to songs produced by oscines, we refer to them hereafter as “songs.”

We measured song structure from recordings of 1,826 individuals from 276 species (see Tobias et al., 2014 for a full data set containing sources and locality information). Recordings came from a number of sources, including the Macaulay Library of Natural Sounds, open‐access online sound archives (e.g., www.xeno-canto.org), commercially available CD/DVDs, and private audio collections of Neotropical ornithologists (see Tobias et al., 2014 for a full data set containing sources and locality information). We selected high‐quality songs, sampled one song per recording (individual) and at least three different individuals per taxon where possible (mean ± *SD*: 6.6 ± 5.4 individuals sampled per lineage).

We extracted five standard core variables from songs (Figure 1) using a custom matlab script code: (1) number of notes in the entire song (note number, *N*), (2) interval between the onset of the first note of the song and the offset of the final note of the song (song duration, D), (3) upper frequency bound of the highest pitched note in the song (maximum song frequency, MaxF), (4) lower frequency bound of the lowest pitched note in the song (minimum song frequency, MinF), and (5) frequency at which the most sound energy was produced (peak frequency, PEAK). From these features, we calculated the rate of note production (N/D, hereafter, “PACE”) and frequency bandwidth (MaxF–MinF).

To examine the predicted trade‐off between the rate at which sounds are produced and the frequency bandwidth of those sounds, we then plotted frequency bandwidth as a function of pace for all individuals for which we had both values (*n* = 1,826). We first used the traditional approach for estimating upper bounds for triangular distributions between two variables (Blackburn, Lawton, & Perry, 1992; Podos, 1997). We binned pace into 2‐Hz increments (0–2 Hz, 2–4 Hz … 38–40 Hz). Within each bin, we chose the song with the maximum bandwidth. We then calculated a linear regression using these maximum values (*n* = 20) to determine the equation for this upper‐bound regression. Sampling limitations inherent in this traditional upper‐bound regression method make it prone to false positives (Wilson, Bitton, Podos, & Mennill, 2013). We therefore used a second analytical method to validate our findings using the more traditional method. We used a sliding binning window to identify the 90th percentile of the frequency distribution data. To avoid sampling error due to outliers, we dropped bins that included fewer than 32 samples. We then used the remaining data to estimate how changes in song pace affected the 90th percentile of the frequency distribution data. Both methods recovered the predicted trade‐off between trill rate and bandwidth (see Results).

To calculate a measure of vocal performance, we used the upper‐bound regression following Podos (1997) and measured the minimum (orthogonal) distance of each song from this regression. This measure is referred to as “vocal deviation” following Podos (2001). Higher values of vocal deviation reflect low vocal performance and lower values reflect high vocal performance (VP; Figure 1), although it is important to note that there has been some questioning of the use of the word “performance” in sexual selection research as performance is a nonneutral term (Kroodsma, 2017). Experimental tests have shown that this measure of vocal performance has biological relevance in a number of species (Ballentine, Hyman, & Nowicki, 2004; Draganoiu, Nagle, & Kreutzer, 2002; Illes, Hall, & Vehrencamp, 2006; Moseley, Lahti, & Podos, 2013; Pasch, George, Campbell, & Phelps, 2011; but see Kroodsma, 2017).

We calculated a mean value for each song variable for each species. Species in this family show little variation in song structure within or between individuals, no repertoires, and low regional variation in song (Tobias et al., 2014); thus, we do not include measures of song variance in our analyses. We log‐transformed all song variables prior to statistical analyses, to meet parametric assumptions of normality and homogeneity of variance. We reduced song variation using phylogenetic PCA (PPCA) (Revell, 2009). Vocal performance was not included in the song PPCA, as it is calculated from variables already included in the PPCA (pace and bandwidth).

### 2.3 Morphological data

We obtained morphological measures (Figure 1) for the same 276 species from museum specimens (see Tobias et al., 2014). To capture morphological variation potentially associated with constraints on song production and modification, we used two variables to represent body size—body mass and tarsus length—the latter being the best univariate index of body size (Freeman & Jackson, 1990). Body mass data were from Dunning (1992). We also measured three beak characters: beak length, measured from the anterior border of the nostril to tip of the beak, and beak width and depth (vertically) at the anterior border of the nostrils. The same person (S. Claramunt) took all beak measurements.

All morphological variables were log‐transformed. We computed body size as the mean of the two log‐transformed body variables and beak size as the mean of the three log‐transformed beak variables. We assumed that overall beak size is related to the trade‐off between force and velocity and that beak dimensions provide a measure of beak moment (indicative of a trade‐off between torque and angular velocity). This allows us to consider the specific effect of angular momentum of the jaw on constraining song modification. The beak's moment of inertia can be defined as the amount of torque required to move the beak at a certain rate of angular acceleration. Beaks with higher moment of inertia will require more torque to move rapidly. We can approximate the beak's moment of inertia as beak width × depth × length, with length to an unknown power. We leave the power unknown because the exact power of length is dependent on beak shape. Thus, we describe the vector parameter of “beak moment” as beak size and log‐transformed beak length (Beak Size, Beak Length; model signal “Beak Size + Beak Length”).

### 2.4 Habitat data

We classified the primary habitat of all lineages using standard published sources (Figure 2). Categories were (1) closed‐canopy forest (“closed”), (2) open‐canopy woodland and shrublands (“semi‐open”), and (3) grasslands and desert (“open”). We used this scoring system to provide an index of habitat structure for each lineage, following standard procedures (Tobias et al., 2014). In the case of generalists, we used literature and published range maps to identify the “primary habitat” as that preferred by the species over the largest geographical area. In practice, classification was simplified by the fact that our habitat categories are broad, with almost all ovenbird species predominantly occurring in one such habitat category.

As an alternative, we treated habitat variation as a continuous variable using bioclimatic data extracted from the geographical range of each species (Seeholzer, Claramunt, & Brumfield, 2017). We gathered 23,588 georeferenced locality records (mean = 79.4 records/species, range = 1–786) representing all study taxa. We obtained the locality records from three general sources: specimens, recordings, and observational records. Specimen records were obtained from ORNIS (www.ornisnet.org). Recording records were obtained from Macaulay Library of Natural Sounds (Cornell Lab of Ornithology) and Xeno‐Canto (www.xeno-canto.org). The coordinates of all documented records (both specimens and recordings) included in this study were vetted for accuracy using gazetteers. The third group of records came from observational data gathered by the eBird citizen science initiative (May 2013 release, Sullivan et al., 2009) which are extensively vetted by expert review (www.ebird.org). To further ensure accuracy, we applied additional filters to the observational records. For each species represented by ten or more localities, we thinned all localities so that no two occurred within 1 km of each other, which is the resolution of the climatic data.

For each locality record, we extracted elevation and 19 bioclimatic variables from the BioClim database of present‐day climatic conditions (Hijmans, Cameron, Parra, Jones, & Jarvis, 2005) and obtained each variable's mean value for all species. To reduce redundancy in the climatic data set, we calculated pairwise Pearson correlation coefficients for the temperature and precipitation variables separately. We retained temperature and precipitation variables that had a Pearson correlation coefficient <0.90 with respect to mean annual temperature (Bio1) and mean annual precipitation (Bio12). Interpretability was increased by purposefully retaining Bio1 and Bio12. We retained four temperature and five precipitation variables: annual mean temperature (Bio1), mean diurnal range (Bio2), isothermality (Bio3), temperature annual range (Bio7), annual precipitation (Bio12), precipitation of driest month (Bio14), precipitation seasonality (Bio15), precipitation of warmest quarter (Bio18), and precipitation of coldest quarter (Bio19). These nine climatic variables were analyzed with the prcomp function in the R Language for Statistical Computing (R‐Core‐Team, 2016). Because the bioclimatic variables were in fundamentally different units for temperature (°C) and precipitation (mm), we used the correlation matrix as opposed to the covariance matrix (Flury, 1997). We used the Kaiser Criterion (eigenvalues greater than one) and retained principal components 1–2, which explained 75% of the climatic variation. Factor loadings, eigenvalues, and percent variance are presented in Appendix S1. For analyses, we retained only the first eigenvector which explained ~60% of the climatic variation (hereafter, “Environment PC1”; Figure 2) because PC2 explained only 14% of the variance and summarized isothermality and precipitation seasonality, which are less generalizable metrics.

### 2.5 Phylogeny

We used a calibrated species‐level phylogeny of the Furnariidae (Figure 2) inferred using three mitochondrial (ND3, CO2, and ND2) and three nuclear genes (RAG‐1, RAG‐2, and Bf7). To calibrate the tree, biogeographic events were used to place priors on the age of the root (split between Tyrannoidae and Furnarioidea of 61 ± 2.8 Ma (Barker, Cibois, Schikler, Feinstein, & Cracraft, 2004)) and on the divergence times of the most recent common ancestor of 12 sets of taxa using two biogeographic events: the closure of the Panamanian Isthmus (3 ± 0.5 Ma following (Weinstock et al., 2005)) and the uplift of the Eastern Cordillera of the northern Andes (3.6 Ma (Gregory‐Wodzicki, 2000) with a 95% age interval of 0.8–16 Ma). We allowed for bidirectional uncertainty in these events. We ran analyses for a total of 150 million generations across seven independent runs. We identified and discarded the burn‐in of each run (total approximately 1 million generations). Converged runs were used to estimate the posterior distribution of topologies and divergence times. We selected the maximum clade credibility (MCC) tree based on a partitioned, Bayesian search of topology and divergence times in BEAST version 1.5.2 (Drummond & Rambaut, 2007). We also sampled 500 trees from the posterior distribution. Details on data collection, phylogenetic inference, as well as the resulting alignment and tree files can be found in Derryberry et al. (2011) and TreeBASE S11550.

### 2.6 Phylogenetic comparative analyses

All phylogenetic comparative analyses were conducted in R 3.3.0 (R‐Core‐Team, 2016). We used phylogenetic generalized least squares models (PGLS) to test the ability of different factors to predict variation in song structure. The dependent factors included the first three principal components from the PPCA used to reduce song variation as well as the individual song traits. The predictors included Environment PC1, Habitat, Body Size, Beak Size, and Beak Moment. We fitted models that included one measure of habitat and one measure of morphology as main factors to reduce issues of collinearity (see below). We analyzed interaction factors between measures of morphology and the categorical measure of habitat, only. We included an interaction term because we predicted that the strength, but not the direction, of the relationship between morphology and song structure may vary across different types of habitats. For example, as habitat becomes more open, and trill rate less limited in acoustic space, a relationship between beak size and trill rate may become more apparent. We include an interaction model only in analyses using categorical measures of habitat variation and not in analyses with habitat treated as a continuous variable as we have no a priori prediction of how particular values of “Environment_PC1” might relate to constraints on song structure. We included a constant model as a point of comparison. One strong outlier was removed from the data set prior to analyses.

Some of the predictors are moderately to highly correlated (λ branch transformation: Beak Size and Beak Moment = 0.85, Beak Size and Body Size *r* = .81, Environment PC1 and Habitat *r* = .72, and Body Size and Beak Moment *r* = .63). Collinearity is common in ecological data sets, and combining or eliminating predictors can underestimate the effects of the included predictor and result in mismodelling the underlying determinants of a given behavior (Freckleton, 2011). We thus model collinear predictors, as AIC information theory methods are generally robust to collinearity. The largest problem arises when one predictor is weak but strongly correlated with a predictor of strong effect—the weak predictor is overestimated and the strong predictor underestimated (Freckleton, 2011). We are thus careful not to overinterpret the effect of weaker correlated predictors. We also minimize effects of collinearity by avoiding stepwise regression (Burnham & Anderson, 2002a) and interactive models between collinear variables (Freckleton, 2011).

The modified GLS approach simultaneously estimates and uses the best branch length transformation to adjust for the degree of phylogenetic nonindependence in the model residuals (Freckleton, Harvey, & Pagel, 2002; Revell, 2010). We used the caper (Orme et al., 2012) library to run PGLS for four models of branch length transformation: Brownian motion (unconstrained random walk), lambda (strength of phylogenetic effects), kappa (speciational change), and delta (exponential accelerating or decelerating change). We used the APE (Paradis, Claude, & Strimmer, 2004) and nlme (Pinheiro, Bates, Debroy, & Sarkar, 2015) libraries for a fifth model, an Ornstein–Uhlenbeck (OU) process (constrained random walk) with a single optimum. We used the MCC tree as our phylogenetic hypothesis. The sample size in all analyses reflects the number of taxonomic units for which we had the appropriate data.

In all model fitting, diagnostic plots were used to check that points on the Q–Q plot approximately fit a straight line and that residual points were randomly scattered. Model fit was evaluated using Akaike Information Criterion corrected for sample size (AICc) (Akaike, 1973; Burnham & Anderson, 2002a). Models greater than two AICc units from the top model (ΔAICc of >2) were considered to have less support, following Burnham and Anderson (2002a). To search for the most parsimonious model, we then removed models within two AICc units of the top model that differed from a higher‐ranking model by the addition of one or more parameters. These were rejected as uninformative, as recommended by Arnold (2010). For traits that we could not identify a most parsimonious model, we averaged the 95% cumulative weight models (including those with uninformative extra terms following Garamszegi (2014)) across a sample of 500 trees from the posterior distribution of trees. We then computed AICc weights for each of the models (1) to determine the total weight of a particular branch length transformation for a particular signal and (2) to determine an average model using the weighted average of the individual model parameters (e.g., the intercept). Parameters are treated as 0 when not present in a given model.

For each song trait, we provide information on the 95% cumulative weight models. We discuss either the most parsimonious model (if one was selected) or the average model with the weight of each signal. We present coefficients (β) and measures of support for models, including model weight (*w*_*i*_), which is the probability that the model of interest is the best model in the set and the evidence ratio in relation to the constant model (ER = *w*_*i*_/*w*_constant model_). For all song traits, we discuss total parameter weights from models fit to the MCC tree. We discuss β from either the most parsimonious model using the MCC tree or from the average model across the posterior distribution, depending on the context. We report the weight of the simplest model as the total weight of the models with a constant signal for the five branch length transformations.

## 3 RESULTS

### 3.1 Song traits

A PPCA on song data yielded three principal components with eigenvalues greater than one. Song frequency measures load strongly onto PC1 (larger values of PC1 indicate lower peak, max and min frequencies, and narrower bandwidth), duration and number of notes load onto PC2 (larger values of PC2 indicate shorter songs with fewer notes), and pace loads onto PC3 (larger values of PC3 indicate faster songs) (Table 1).

**Table 1:** Eigenvalues and loadings of song traits on principal components (PC) from PPCA. Significant loadings in bold

Trait	Song PC1	Song PC2	Song PC3
Peak frequency	**−0.97**	−0.08	0.05
Frequency bandwidth	**−0.70**	0.00	−0.46
Maximum frequency	**−0.98**	−0.07	−0.11
Minimum frequency	**−0.73**	−0.11	0.40
Song duration	0.14	**−0.79**	−0.48
Number of notes	0.14	**−0.96**	−0.01
Pace	−0.02	−0.49	**0.71**
Eigenvalues	2.98	1.82	1.23

Using the 90th percentile method, we found support for the predicted trade‐off between pace and bandwidth, such that as song pace increases, songs are more limited in bandwidth (slope = −15.59, *y*‐intercept = 3,034, *F*_1,36_ = 68.2, *p* = 8 × 10^−10^, and *R* ^2^ = 0.64). The upper bound describing this trade‐off was *y* = −79.374*x* + 5066.2 (*R* ^*2*^ = .55).

### 3.2 Beak morphology, habitat structure, and signal design

We report AICc for all signals and all branch length transformations (Appendix S2). For all song traits, we provide AICc, model weight, and ER for the 95% cumulative weight models (Appendix S3) (Burnham & Anderson, 2002b). We also report the top model and models within two AICc (Table 2) and their coefficients of variation (Appendix S4), dropping models with uninformative parameters (Anderson & Burnham, 2002). Finally, we provide the parameter total weights (Table 3) and coefficients of variation averaged across the posterior distribution of trees (Table 4) (Garamszegi, 2014).

**Table 2:** Top model and models within two AICc (models with uninformative terms dropped) reported for each of the three principal components (PC) describing song structure and for individual song traits, which are grouped with the PC on which they loaded most strongly. Values reported for the best branch length transformation

Trait	Signal	ΔAICc^a^	w^b^	ER^c^
Song PC1	Body Size		0.45	178
PEAK	Body Size		0.42	581
BW	Habitat		0.29	6
MAXF	Body Size		0.46	99
MINF	Body Size + Habitat		0.36	6,179
MINF	Beak Size + Habitat	1.44	0.18	3,001
Song PC2	Constant		0.13	0
DUR	Beak Size		0.17	2
DUR	BODY size	1.53	0.08	1
NN	Habitat		0.19	8
Song PC3	Beak Moment + Habitat		0.13	24,364
Song PC3	Body Size + Habitat	0.23	0.12	21,681
Song PC3	Beak Moment	0.75	0.09	16,729
Song PC3	Beak Size + Habitat	1.89	0.05	9,489
PACE	Beak Size + Habitat		0.14	2,411
PACE	Body Size + Habitat	0.55	0.11	1,831
VP	Beak Moment × Habitat		0.48	10,558

AIC, Akaike Information Criterion; PEAK, song peak frequency, BW, song frequency bandwidth, MAXF, song maximum frequency, MINF, song minimum frequency, DUR, song duration, NN, number of notes, PACE, song pace, and VP, vocal performance score.

^a^model AICc—top model AICc, ^b^model weight, and ^c^evidence ratio.

**Table 3:** For all song traits, parameter total weights. Parameters included in competitive models are in black. See Table 2 for abbreviations

	Beak size	Habitat (semi‐open)	Habitat (open)	Beak length	Beak Size × Habitat (semi‐open)	Beak Size × Habitat (open)	Beak Length × Habitat (semi‐open)	Beak Length × Habitat (open)	Body Size	Body Size × Habitat (semi‐open)	Body Size × Habitat (open)	Environment PC1
Song PC1	0.234	0.232	0.232	0.096	0.008	0.008	0.003	0.003	0.762	0.032	0.032	0.209
PEAK	0.281	0.168	0.168	0.120	0.005	0.005	0.001	0.001	0.718	0.016	0.016	0.256
BW	0.276	0.824	0.824	0.082	0.074	0.074	0.029	0.029	0.350	0.144	0.144	0.079
MAXF	0.171	0.229	0.229	0.055	0.007	0.007	0.002	0.002	0.820	0.050	0.050	0.239
MINF	0.484	0.796	0.796	0.214	0.048	0.048	0.007	0.007	0.516	0.053	0.053	0.081
Song PC2	0.358	0.449	0.449	0.085	0.095	0.095	0.009	0.009	0.263	0.095	0.095	0.151
DUR	0.669	0.152	0.152	0.184	0.011	0.011	0.001	0.001	0.224	0.004	0.004	0.229
NN	0.340	0.904	0.904	0.073	0.083	0.083	0.006	0.006	0.311	0.100	0.100	0.038
Song PC3	0.713	0.662	0.662	0.507	0.096	0.096	0.037	0.037	0.287	0.056	0.056	0.119
PACE	0.711	0.771	0.771	0.219	0.137	0.137	0.023	0.023	0.289	0.079	0.079	0.083
VP	0.878	0.712	0.712	0.620	0.549	0.549	0.532	0.532	0.122	0.113	0.113	0.156

**Table 4:** For all song traits, β values for all parameters averaged across a sample of 500 trees from the posterior distribution of trees. Parameters included in competitive models are in black. See Table 2 for abbreviations

	Intercept	Beak Size	Habitat (semi‐open)	Habitat (open)	Beak Length	Beak Size × Habitat (semi‐open)	Beak Size × Habitat (open)	Beak Length × Habitat (semi‐open)	Beak Length × Habitat (open)	Body Size	Body Size × Habitat (semi‐open)	Body Size × Habitat (open)	Environment PC1
Song PC1	−8.537	1.876	−0.009	−0.010	−0.299	0.000	0.000	0.000	0.000	5.205	0.115	0.145	−0.001
PEAK	3.677	−0.050	−0.002	−0.002	0.008	0.000	0.000	0.000	0.000	−0.105	0.000	0.000	0.000
BW	3.192	0.002	0.055	−0.001	−0.005	−0.039	−0.033	0.018	0.018	−0.008	−0.030	−0.038	−0.000
MAXF	3.743	−0.017	0.003	0.004	0.001	0.000	0.000	0.000	0.000	−0.099	−0.004	−0.006	−0.000
MINF	3.571	−0.115	−0.021	0.011	0.019	0.003	0.003	0.000	0.000	−0.085	0.002	0.000	0.000
Song PC2	−0.478	0.242	−0.409	−2.584	0.141	0.164	1.336	−0.065	−0.118	0.095	0.116	0.865	0.008
DUR	−0.028	0.387	0.005	0.004	−0.018	0.000	0.000	0.000	0.000	0.078	0.000	0.000	−0.000
NN	1.377	−0.054	0.140	0.331	−0.001	−0.011	−0.075	0.000	0.000	−0.045	−0.026	−0.077	0.000
Song PC3	7.134	−9.325	−0.064	1.700	2.919	−0.388	−0.854	0.264	0.159	−2.059	−0.034	−0.174	0.002
PACE	1.574	−0.558	0.098	0.224	0.040	−0.021	−0.079	0.000	0.000	−0.177	−0.022	−0.039	0.000
VP	30.530	−0.841	−18.543	−31.088	2.210	27.971	26.104	−16.535	−11.540	1.690	10.561	17.217	0.002

**Figure 3:**
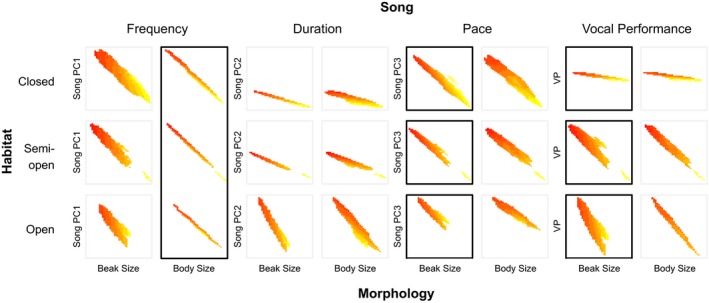
Diversification of song traits is differentially impacted by habitat and morphology. Plotted values indicate the region of predicted song trait values (Song PC1–3 and vocal performance) for 95% of the observed morphological measurements (beak size and body size) within each habitat type (closed, semi‐open, and open). All song traits increase along the *y*‐axis (i.e., larger *y*‐values indicate higher frequency, longer duration, faster pace, higher performance). Labels of column pairs indicate song traits that loaded most strongly onto Song PCs (e.g., frequency traits on Song PC1). Heat maps indicate variation in beak length as a third axis to convey information about predicted beak moment (larger values are more yellow). Informative relationships (ΔAIC < 2 of top model; parameter weights >30%) are indicated in black boxes. Left to right: (Frequency) Larger birds sing lower frequency songs. (Duration) No informative relationships. (Pace) Songs in open habitats are faster, and birds with greater beak moment sing slower songs. (Vocal Performance) Birds with greater beak moment sing lower performance songs, especially in open habitats. AIC, Akaike Information Criterion

As predicted, body size best explained variation in spectral characteristics of song. The most parsimonious model for Song PC1 was Body Size under the λ branch length transformation and garnered 45% of the model weight (Table 2). All remaining models individually had less than 16% of the total weight. As a parameter, Body Size had 76.2% of the weight across all candidate models, providing strong support for this parameter explaining variation in song spectral characteristics. Birds with larger bodies sang lower frequency songs (ER > 178, β = 6.58 units of Song PC1/unit of Body Size; Figure 3). We found only weak support for other morphological or environmental parameters explaining variation in Song PC1 (parameter total weights: Habitat = 23.2%, Environmental PC1 = 20.9%, Beak Size = 23.4%, Beak Moment = 9.6%).

Our findings for individual song spectral traits were generally consistent with results for Song PC1. Body Size received strong support as the most parsimonious model for peak frequency (ER > 581; *w*_*i*_ = 0.42) and maximum frequency (ER > 99; *w*_*i*_ = 0.46). In addition, the total weight for Body Size as a parameter was high for most spectral traits (PEAK: 71.8%, MAX: 82%, MIN: 51.6%) except bandwidth (35%). However, we did find evidence of a role for habitat in explaining some variation in spectral features of song but not in the direction predicted under sensory drive. We found strong evidence that additive models of morphology and habitat explained variation in minimum frequency (average model: Habitat + Beak Size + Body Size), with Habitat garnering 80% of the total weight of candidate models. Counter to the prediction of lower minimum frequencies in more closed habitats, we found that minimum frequency was lower in semi‐open habitats than in either open (β = 0.03) or closed (β = 0.02) habitats. Minimum frequency did vary as predicted with beak and body size, such that larger birds (B = −0.09) with bigger beaks (β = −0.12) sing lower minimum frequencies. For song bandwidth, habitat was the most parsimonious model but with a low evidence ratio (ER < 6.5). Again, counter to the prediction of reduced bandwidth in more closed habitats, we found narrower bandwidth songs in more open habitats (β = −0.07). Overall, we find that spectral features of song vary with body size, and any association with habitat is not consistent with predictions under sensory drive.

We did not have a priori predictions under the sensory drive or magic traits hypotheses regarding song length or number of notes and did not find strong evidence of habitat or morphology explaining variation in these two features of song (all ER < 8). The most parsimonious model for Song PC2 was the simplest model under the delta branch length transformation. We also found weak evidence for the most parsimonious models for both song length (ER < 2.5) and number of notes (ER < 8). An average model for song length includes both Beak Size and Body Size, with Beak Size receiving more weight than Body Size (Beak Size *w*_*i*_ = 0.67 and Body Size *w*_*i*_ = 0.22), such that song length increases with beak size (β = 0.39). An average model for number of notes includes Habitat, Body Size, and Beak Size, but Habitat receives higher weight than either morphological parameter (Habitat = 0.90, Beak Size = 0.34, Body Size = 0.31), such that the number of notes in a song increases as habitats become more open (Habitat semi‐open β = 0.14, Habitat open β = 0.33). However, consistently low evidence ratios for traits associated with Song PC2 indicate high model selection uncertainty.

Consistent with predictions, we found that both morphology and habitat explain variation in song pace. For Song PC3, we found strong evidence for top additive models including the parameters Beak Size, Beak Length, Body Size, and Habitat (ER range: 9,489–24,364). Considering the average additive model, Beak Size received the highest weight (71.3%) followed by Habitat (66.2%) and Beak Length (51%). However, Body Size has low parameter weight (28.7%), and considering the known effect of collinearity on model selection (Freckleton, 2011), it is unlikely that Body Size is an important factor explaining variation in Song PC3. Because we approximate beak moment as a vector parameter (Beak Size, Beak Length), an average additive model that includes these two terms is effectively beak moment. Therefore, we find that birds with larger beak moment produce songs with lower values of Song PC3 (i.e., slower songs; Beak Size β = −9.33, Beak Length β = 2.92), and birds in more open habitats produce songs with higher values of Song PC3 (i.e., faster songs; Habitat open β = 1.7) (Table 3). Given the collinearity of beak size and beak length, we plotted the region of predicted Song PC3 values for 95% of the observed beak measurements within each habitat type to inspect the direction of the relationship (Figure 3). We constructed this plot for an approximation of beak moment as beak width × depth × length^2^. We also checked against an approximation with length^3^ and noted very little difference in predicted relationships.

Considering that pace loaded most strongly onto Song PC3, we found similar results for pace. For pace, we found strong evidence for the most parsimonious models of Beak Size + Habitat (ER > 2,411) and Body Size + Habitat (ER > 1,831). Considering how these parameters contributed to an average model, Habitat had the highest weight (77.1%) followed by Beak Size (71.1%). Again, Body Size received very low total parameter weight (28.9%) and as such is probably not important. Song pace is faster in more open habitats (Habitat semi‐open β = 0.98, Habitat open β = 0.22), and birds with larger beaks (β = −0.56) produced slower songs (Table 3).

Consistent with predictions, beak moment best explained variation in vocal performance, but unexpectedly, the strength of this relationship varied across habitats. There was strong evidence that the most parsimonious model included an interaction between Beak Moment and Habitat (ER > 10,558) with an OU branch length transformation. The total weight of the model was 48%, and all other models individually had less than 21% of the total weight. Beak and habitat parameters had similar weights across models (Beak Size = 87.8% and Beak Length = 62%, Habitat = 71.2%). Birds with larger beak moment (as we have approximated it) produced lower performance songs (beak size β = 0.84, beak length β = 2.2). Birds in more open habitats produce higher performance songs (Habitat semi‐open β = 18.5, open β = 31.1). The relationship between beak moment and vocal performance is strongest in open habitats, and weakens as habitat becomes more closed. Given the collinearity of beak size and beak length, we plotted the region of predicted vocal performance values for 95% of the observed beak measurements within each habitat type to inspect the direction of the relationships (Figure 3).

Finally, we found little support for the continuous measure of habitat (Environment PC1) explaining variation in any of the song traits. Environment PC1 did not garner more than 25% of the total weight for any individual song trait or for any of the Song PCs (Table 3). Habitat (categorical measure) had high weight as a parameter for a number of song traits, and yet Environment PC1 did not.

## 4 DISCUSSION

We have shown that variation in ovenbird songs arises through a combination of direct selection on signal design via transmission properties of the environment and indirect selection on song characters as a byproduct of selection on morphological traits associated with diversification into different ecological niches. Indirect selection is the primary force shaping spectral features of song, whereas both direct and indirect selection act on song tempo and performance. Together, these findings suggest that ecological selection on morphology indirectly drives the evolution of songs in ovenbirds, whereas habitat structure mediates the strength of indirect selection on song tempo and performance.

### 4.1 Magic traits

We found strong evidence that multiple magic traits influence the diversification of most song traits in ovenbirds. Body size was the most important parameter for spectral features of song, whereas beak size was more important for temporal and performance features. These results make sense because body size is primarily thought to affect sound production, specifically the frequencies of sound which birds can produce efficiently, whereas beak size is primarily thought to affect sound modification.

Our analyses indicated that larger birds produce songs at lower peak, maximum and minimum frequencies, in agreement with previous empirical studies (Mason & Burns, 2015; Ryan & Brenowitz, 1985; Seddon, 2005; Tubaro & Mahler, 1998; Wallschäger, 1980) and consistent with the traditional understanding of how birds produce sound (Nowicki & Marler, 1988). Thus, the diversification of ovenbird body size has contributed to the diversification of spectral components of their song.

We also found that temporal and performance features of ovenbird song correlate with beak size, such that birds with larger beak size produce slower paced songs at lower performance. Our findings agree with other studies showing an effect of beak size on the pace of sound production (Huber & Podos, 2006; Seddon, 2005) and on the performance of a trade‐off between song pace and bandwidth (Ballentine, 2006; Huber & Podos, 2006; Podos, 2001), including in the woodcreepers, a subclade of the ovenbird family, as currently defined (Derryberry et al., 2012). Specifically, we find that larger beak size is associated with slower paced and lower performance songs. Thus, our results suggest that as ovenbirds have diversified in beak size, they have also diversified in some temporal and performance components of song.

Birds with larger beaks are thought to face a limitation on producing high‐performance songs because of a trade‐off between force and velocity. This idea has been examined—and supported—extensively in Darwin's finches (Herrel et al., 2008; Podos & Nowicki, 2004a). In these finches, species with larger beaks have more developed musculature allowing them to crack larger seeds, an increased capacity for force that trades off with velocity, such that larger beaked birds are only able to open and close their beaks relatively slowly. However, this trade‐off between force and velocity seems less likely to constrain song production in ovenbirds, most of which are specialist insectivores (Wilman, Belmaker, Simpson, De La Rosa, & Rivadeneira, 2014) with beak musculature adapted to softer food items. Small seeds are only thought to make up >20% of the diet in five ovenbird species (*Geositta punensis*,* G. antarctica*,* Asthenes dorbignyi*,* A. arequipae*, and *A. huancavelicae*) (Wilman et al., 2014). In support of the alternative idea that song modification may be constrained by the angular momentum of the jaw (Palacios & Tubaro, 2000), we found evidence that beaks with higher moment of inertia (as we approximated it) are more limited in vocal performance, such that ovenbirds with larger beak moment produce slower songs with lower performance. Our findings do not rule out effects of force and velocity but suggest that diversification in features of the beak that affect angular momentum of the jaw may constrain song diversification.

The key mechanism underlying “magic trait” speciation (Gavrilets, 2004; Thibert‐Plante & Gavrilets, 2013) is the linkage between a trait used to recognize mates (here, songs) and a trait under ecological selection (here, beak and body size). When we consider body size and beak size as predictors in our models, we find that morphology correlates strongly with variation in song pitch, pace, and performance. As birds diversify in body size, we expect that spectral features of song will also diversify. Thus, body size has the potential to act as a magic trait whether birds are increasing or decreasing in size. For beak size, the effect is not consistent across the size axis. Specifically, birds with larger beaks sing lower, slower songs at a lower vocal performance, whereas birds with small beaks produce more variable songs ranging from low, slow songs to high, fast songs. Any increase in beak size may lead to song divergence from ancestral lineages, with beak size then acting as a magic trait. Although it is less certain that decreases in beak size would necessarily lead to signal divergence, such decreases may remove constraints on sound modification, allowing songs to diverge into new acoustic space (e.g., higher, faster songs). Although beaks have the potential to act as magic traits, the effect of beak diversification on signal divergence depends in part on whether beaks increase or decrease in size.

Our finding that morphological traits are correlated with spectral, temporal, and performance traits of ovenbird songs suggests that diversification in body and beak size could have led to correlated divergence in mating signals, thereby strengthening reproductive isolation among lineages (Derryberry et al., 2012). However, the extent to which particular song traits affected by morphological divergence also function in mate recognition remains unclear. Future research should test which specific song traits (e.g., performance and peak frequency) are salient in mate recognition in ovenbirds.

### 4.2 Sensory drive

The sensory drive hypothesis is widely accepted on the basis of case studies across a number of different animal groups (Cummings, 2007; Endler, 1992, 2000; Wiley & Richards, 1982), yet its relevance across larger samples of species has been questioned, particularly in birds (Boncoraglio & Saino, 2007; Ey & Fischer, 2009). We found that our categorical measure of habitat was a competitive model for song bandwidth, minimum frequency, number of notes, pace, and vocal performance. However, the direction of the relationship between habitat and song variation for the first two song traits was opposite that predicted under sensory drive, whereas the relationship with the other three traits was consistent with predictions from sensory drive (Tables 2 and 4). Although there was low model support for note number, habitat was clearly an important parameter explaining variation in the number of notes in a song, with birds in more open habitats having more notes. Altogether, these results suggest that the influence of direct ecological drivers of song divergence in ovenbirds is limited to temporal components of song structure (song pace and potentially number of notes), which also influence song performance. In the context of previous studies, our findings suggest that habitat can drive song divergence, at least in temporal characters.

Although our findings suggest that habitat‐dependent selection has not acted on the spectral components of song in ovenbirds, we note that songs in this family fall mainly within the ideal frequency transmission window for most habitats (Wiley & Richards, 1982). Minimum frequencies of ovenbird songs are above 1 kHz, and the peak and maximum frequencies of most songs are lower than 4–5 kHz, thus occupying the band of intermediate frequencies (1–4 kHz) that do not suffer much variation in attenuation between habitats (Linskens et al., 1976). While direct ecological selection may have played a role in limiting the overall frequency range of ovenbird songs, it is not possible to determine whether sensory drive has selected against songs outside this frequency band, or alternatively whether song phenotypes have not diversified completely into potential acoustic space (e.g., frequencies above 5 kHz in open habitats) because of morphological constraints or conservatism of ancestral traits. However, it seems plausible that restriction to the ideal frequency window limits the strength of habitat‐mediated sensory drive on spectral components of song in ovenbirds.

Although habitat as a categorical measure was an important parameter for a number of song traits, our continuous measure of habitat (Environment PC1) did not help to explain variation in any feature of song. Our categorical habitat scores and Environment PC1 were correlated, and the fact that associations with song were weakened when using a continuous variable underscores our general finding that direct environmental effects on song structure are relatively limited.

### 4.3 Interactions among mechanisms

Our findings suggest that variation in the signaling environment and constraints on sound modification act independently on song pace. In contrast, we found evidence of both direct and indirect selection interacting to explain divergence in song performance. A measure of the trade‐off between torque and velocity (beak moment) was the most important parameter fitted to vocal performance, yet this relationship varied across habitats, such that vocal performance was more sensitive to increases in beak moment in more open habitats. These findings confirm that strong interactions between habitat and morphology are fundamental in governing the magnitude and direction of song divergence and thus suggest that direct and indirect mechanisms of signal evolution cannot be considered in isolation.

### 4.4 Stochasticity

Our phylogenetic comparative analyses suggest that shared ancestry and stochastic processes explain a large component of song evolution in ovenbirds, consistent with previous findings that evolutionary age explains a large proportion of song divergence in the family (Tobias et al., 2014). However, we are able to rule out the possibility that stochasticity alone explains the diversification of most song traits in our study. The main exceptions are song length, number of notes, and bandwidth. We did not have a priori expectations that song length and note number would vary with morphological traits or habitat structure, although we did expect that song bandwidth would decrease in more closed habitats and for birds with larger beaks. For all three of these song traits, the evidence for fitted models was very low. Moreover, we only explored parameters associated with ecological selection on these traits, and thus, we may have overlooked a role for social or sexual selection, particularly as song length, note number, and bandwidth have all been shown to be under sexual selection via mate choice in other species (reviewed in Andersson, 1994; Catchpole & Slater, 2008; Searcy & Andersson, 1986).

## 5 CONCLUSIONS

Two deterministic processes—sensory drive and correlated evolution—shape acoustic signals in ovenbirds. These pathways of song divergence act both independently and in concert, with ecological selection on beak and body size playing the most widespread role. Although body size is particularly important in explaining how spectral features of song evolve and beak size is important in explaining how temporal features of ovenbird songs evolve, morphology alone is not the best predictor. Key temporal and performance measures of song are best explained by both beak size and habitat. Thus, we conclude that a combination of sensory drive and correlated evolution drives signal evolution, with the outcome tightly linked to ecology. In addition, we have demonstrated separate roles for body size and beak size via their constraints on both signal production and signal modification, respectively, providing new evidence that different potential “magic traits” can have contrasting effects on signal diversification. Our work highlights the importance of both direct and indirect sources of ecological selection as critical factors that need to be considered together in models of mating signal evolution.

## CONFLICT OF INTEREST

None declared.

## AUTHOR CONTRIBUTIONS

EPD, NS, and JAT conceived the study. EPD, NS, SC, GFS, RTB, and JAT provided data. EPD, GFS, and GED analyzed data. EPD and JAT wrote the manuscript. All authors contributed to revisions.
